# 

**Published:** 1890-10

**Authors:** 


We are under obligations to the respective authors for the following Treatises and Essays :

IDENTITÉ DE LA DENGUE ET LA GRIPPE-INFLUENZA.—Par le Docteur Jules Rouvier, Professeur de Clinique Obstetricale et Gynécologique à la Faculté Française de Médecine de Beyrouth, etc., etc

STRICTURE OF THE RECTUM.—A study of ninety-six cases, with illustrations, by Charles B. Kelsey, M. D., Professor N. Y. Post-Graduate School, etc.

VARICOCELE.—By Thomas W. Kay, M. D., late Surgeon Johanniter Hospital, Beyrout, Syria.

CANCER; ITS CAUSES AND TREATMENT.—By G. H. Stockham, M.D., Oakland, Cal.

A CASE OF CORNEAL TRANSPLANTATION FROM THE RABBIT'S TO THE HUMAN EYE, etc.—By William F. Smith, M. D., Oculist and Aurist to the Emergency Hospital, Chicago, etc.

FIVE CASES OF VAGINAL HYSTORECTOMY.—By W. F. McNutt, M. D., Prof. University of California.

AN EXPLANATION OF THE PHENOMENA OF IMMUNITY AND CONTAGION, based upon the action of Physical and Biological Laws.—By J. W. McLaughlin, M. D., Austin, Tex.

SEWERAGE.—An address by Col. George E. Waring, Jr., Columbus, O.

BOSTON UNIVERSITY SCHOOL OF MEDICINE. Eighteenth Annual Announcement, etc., 1890.

---

THE MONIST, is the name of a NEW QUARTERLY, to be published by the Open Court Publishing Co., of Chicago, to be commenced October 1st.

